# Minority stress, social support and mental health among lesbian, gay, and bisexual college students in China: a moderated mediation analysis

**DOI:** 10.1186/s12888-023-05202-z

**Published:** 2023-10-13

**Authors:** Huijun Li, Xiaoling Liu, Qingyong Zheng, Siyuan Zeng, Xiaofeng Luo

**Affiliations:** 1 School of Public Health, Lanzhou University, Lanzhou, China; 2 Wenzhou Center for Disease Control and Prevention, Wenzhou, China; 3 Evidence-Based Medicine Center, School of Basic Medical Sciences, Lanzhou University, Lanzhou, China; 4 The First School of Clinical Medicine, Lanzhou University, Lanzhou, China

**Keywords:** LGB, School bullying, Minority stress, Mental health, Perceived social support

## Abstract

**Background:**

The existing body of research exploring minority stressors and their impact on the mental health of Lesbian, Gay, and Bisexual (LGB) students in China remains limited in scope and often restricted to specific geographic regions..

**Methods:**

A combination of snowball and targeted sampling strategies was used to recruit lesbian, gay and bisexual students (*N* = 1,393) for a cross-sectional, online survey in China. Participants (Mage = 20.00 years; 60.23% assigned male at birth) were tasked with completing a comprehensive questionnaire designed to capture various dimensions, including gender expression, minority stressors (e.g., school bullying, internalized homophobia), social psychological resources (e.g., perceived social support), and mental health-related outcomes (e.g., depression, anxious and stress). Our analytical approach involved hierarchical multiple regression analyses, mediation and moderated mediation modeling to elucidate the intricate interplay among these factors.

**Results:**

Our findings shed light on the pronounced mental health disparities afflicting LGB college students in China, with notable prevalence rates of depression (48.1%), anxiety (57.1%), and stress (37.5%). A significant positive correlation was observed between experiences of school-based victimization and internalized homophobia, which, in turn, exhibited a direct association with affective symptoms.School bullying was positive with internalized homophobia, which was positively associated with affective symptoms.In addition to unveiling the indirect effects of school bullying on affective symptoms, our study identified direct links in this complex relationship. Notably, the availability of social support emerged as a pivotal factor, serving as a moderator within the mediation model by mitigating the path from school-based victimization bullying to internalized homophobia (β = -0.077, *P* = 0.040).

**Conclusions:**

This study underscores the pervasive and concerning mental health disparities experienced by LGB college students in China. In response, institutions of higher learning should intensify anti-bullying initiatives tailored to LGB students and implement comprehensive gender education programs. Moreover, concerted efforts should be directed at enhancing the accessibility of social support resources for LGB college students, with the aim of cultivating and sustaining favorable psychological well-being.

**Supplementary Information:**

The online version contains supplementary material available at 10.1186/s12888-023-05202-z.

## Introduction

In recent decades, research attention has increasingly turned toward the mental well-being of sexual minorities [1]. Studies have shown that, comparedto heterosexual individuals, lesbian, gay, bisexual (LGB) individuals face atwo-fold greater risk of experiencing mood and anxiety disorders [2]. However, much of the research on LGB stress and coping in China, as in other countries, has predominantly focused on adults [3], with limited attention given to student populations.

### Minority stress theory

The conceptual framework known as Minority stress theory provides valuable insights into understanding the mental health disparity by sexual minorities. This theory posits that sexual minorities are at an elevated risk of psychological distress due to their encounters with external prejudice events, discrimination and internal stress processes such as internalized homophobia and concealment [4, 5]. A substantial body of evidence consistently supports this theory within the LGB community, underscoring how experiences like discrimination, victimization, bullying, concealment, and internalized homophobia contribute to psychological distress and stress-related conditions, including suicidal ideation [6, 7]. Notably, LGB individuals are particularly susceptible to daily social stress stemming from discrimination based on their sexual orientation [8]. The model also suggests that distal stressors, such as school bullying, can be internalized into the proximal stressors, like internalized homophobia [9]. Furthermore, minority stress model posits that the relationship between stressors related to sexual and gender minority statusand mental health outcomes may be moderated by social support and coping strategies [5]. Numerous studies have found links between social support and the health of LGBT individuals [10]. While social support is often seen as an interpersonal phenomenon, research has revealed both direct and stress-buffering effects on mental health [10].

### Current situation of China

However, much of this evidence has been established primarily in Western countries, which raises questions about the universal applicability of this theory in understanding and addressing mental health challenges among LGB individuals globally. In the context of China, there is a relative scarcity of literature on minority stress theory, particularly concerning Chinese LGB students. Moreover, there has been limited investigation into school bullying and its association with mental health issues like depression, anxiety, and stress symptoms [3, 11].

China places significant emphasis on social bonds and interdependent relationships within society [1]. Schools, as environment for socialization, play a critical role in shaping students' psychosocial well-being [12]. The college years, in particular, represent a high-risk period for the development of mental health concerns among all students [13]. Unfortunately, schools can sometimes become hostile environments, especially for students who are more likely to experience passivity. LGB students may face additional pressures compared to their heterosexual counterparts, including the heightened risk of experiencing school bullying, which significantly elevates their susceptibility to poor mental health outcomes [14, 15]. Identifying as a sexual minority can increase a person's chances of suffering from bullying at school [16]. In China, where non-heterosexuality remains stigmatized and less discussed [17], social disapproval of sexual identity often compels many LGB individuals to conceal their sexual orientation [3]. UNESCO (2013) defines homophobia as the underlying attitude that fuels homophobic bullying and encompasses the fear of, rejection of, or aversion to gender and sexual minority [16]. The intersection of sexual prejudice and cultural norms in China may further expose LGB students to minority stress, increasing their vulnerability to mental health challenges.

### The current study

This study endeavors to address several gaps in the current body of literature concerning school bullying, internalized homophobia, social support, and mental health within the context of Chinese LGB students. Our research aims to explore affective symptoms among LGB college students in China and examine how minority stress factors influence their mental health. To achieve this, we employ hierarchical multiple regression analyses and structural equation model (SEM).

## Methods

### Procedure

Data collection for this study was conducted through an Internet-based survey. We employed a combination of snowball and targeted sampling methods to recruit participants. A thematic poster containing questionnaire links was created and disseminated through the “WeChat” and “QQ group of Rainbow Wall” – popular online communities among LGBT students. This recruitment strategy spanned 142 colleges and universities, encompassing 31 provinces in China.

Participants were encouraged to forward the study information to potentially eligible individuals and groups. Recognizing the challenges of recruiting bisexual women and LGB individuals, we specifically targeted advertising efforts towards platforms such as “The L” and “Lesfun” APP, which are known for their relevance to these groups.

Prior to questionnaire completion, participants were directed to a web-based information statement outlining the study's purpose: “to gain a better understanding of the health status of lesbian, gay, and bisexual.” This statement also delineated the participation criteria (college student; self-identified as LGB; residing in China), associated risks and benefits, and a confidentiality agreement. Participants who consented to participate proceeded to completed the questionnaire online using the “wenjuanxing” platform, with each participant receiving a $1 incentive.

### Participants

A total of 1,678 individuals participated in the survey.After excluding those who did not meet the inclusion criteria (including none of the main study variables (*n* = 1), non-college students (*n* = 7), individuals younger than 15 (*n* = 5), duplicate Internet Protocol addresses (*n* = 2), those identitying as straight and transgender (*n* = 28), and respondents providing fraudulent data on the scales (*n* = 131)), 1,504 participants were retained.

To identify and address multivariate outliers, the Mahalanobis distance was calculated. Outliers were removed for the following variables: school bullying (42 cases), mental health (46 cases), social support (22 cases), and internalized homophobia (1 cases). In total, 111 cases were identified as multivariate outliers and were excluded from subsequent analyses, resulting in a final analytical sample of 1,393 participants.

The age of participants ranged from 15 to 31 years, with a median age of 20.00 years old (interquartile range: 19.00–21.00). Among the participants, 60.23% were assigned male at birth, while 20.4% identified as lesbian, 48.4% as gay, and 31.2% as bisexual. Additionally, 38.4% resided in rural area, and 9.2% held a master's degree or higher. Further demographics details for the entire sample and by gender are presented in Table 1.

**Table 1:** Demographics of the participants

	Total(*n* = 1393) N(%)/Mean(S.D.)	Male(*n* = 839) N(%)/Mean(S.D.)	Female(*n* = 554) N(%)/Mean(S.D.)	χ^2^/t-vlue	*P*-value
Age(years)					
≤ 20	780(55.99)	454(54.11)	326(58.84)	3.033	0.082
> 20	613(44.01)	385(45.89)	228(41.16)		
Sexual orientation					
Gay/Lesbian	959(68.84)	675(80.45)	284(51.26)	132.545	< 0.001
Bisexual	434(31.16)	164(19.55)	270(48.74)		
Ethnicity					
Han	1294(92.89)	778(92.73)	516(93.14)	0.086	0.770
Ethnic minorities	99(7.11)	61(7.27)	38(6.86)		
Urban or rural areas					
Urban	858(61.59)	449(53.52)	409(73.83)	58.187	< 0.001
Rural	535(38.41)	390(46.48)	145(26.17)		
Education					
Freshmen	365(26.20)	191(22.77)	174(31.41)	16.654	0.002
Sophomore	332(23.83)	218(25.98)	114(20.58)		
Junior	341(24.48)	201(23.96)	140(25.27)		
Senior	227(16.30)	145(17.28)	82(14.80)		
Master and above	128(9.19)	84(10.01)	44(7.94)		
Major					
Humanities and Social sciences	532(38.19)	262(31.23)	270(48.74)	55.646	< 0.001
Science, engineering and agriculture	702(50.39)	490(58.40)	212(38.27)		
Medical	159(11.41)	87(10.37)	72(13.00)		
Way of living					
School dormitory	1211(86.93)	738(87.96)	473(85.38)	2.525	0.283
Share with others outside school	146(10.48)	83(9.89)	63(11.37)		
Living alone outside school	36(2.58)	18(2.15)	18(3.25)		
Parents' marital status					
Stability	1132(81.26)	700(83.43)	432(77.98)	7.377	0.025
Instability	68(4.88)	33(3.93)	35(6.32)		
Divorced or bereavement	193(13.85)	106(12.63)	87(15.70)		
Mother's education level					
Middle school and below	669(48.03)	474(56.50)	195(35.20)	60.633	< 0.001
High school and above	724(51.97)	365(43.50)	359(64.80)		
Father's education level					
Middle school and below	571(40.99)	399(47.56)	172(31.05)	37.600	< 0.001
High school and above	822(59.01)	440(52.44)	382(68.95)		
monthly expense					
≤ 1000	124(8.90)	76(9.06)	48(8.66)	11.378	0.010
1001 ~	916(65.76)	576(68.65)	340(61.37)		
2001 ~	259(18.59)	141(16.81)	118(21.30)		
3001 ~	94(6.75)	46(5.48)	48(8.66)		
Tobacco use					
Yes	108(7.75)	47(5.60)	61(11.01)	13.650	< 0.001
No	1285(92.25)	792(94.40)	493(88.99)		
Alcohol use					
Yes	596(42.79)	337(40.17)	259(46.75)	5.909	0.015
No	797(57.21)	502(59.83)	295(53.25)		
Suicide attempts					
Yes	80(5.74)	62(7.39)	18(3.25)	10.568	0.001
No	1313(94.26)	777(92.61)	536(96.75)		
Self-rated esteem					
Agree	1239(88.94)	758(90.35)	481(86.82)	5.207	0.074
Neutral	135(9.69)	73(8.70)	62(11.19)		
Disagree	19(1.36)	8(0.95)	11(1.99)		
Self-rated health					
Healthy	1058(75.95)	649(77.35)	409(73.83)	4.841	0.089
Fair	297(21.32)	173(20.62)	124(22.38)		
Unhealthy	38(2.73)	17(2.03)	21(3.79)		
Depression symptoms	10.58(8.90)	10.2(8.44)	11.16(9.52)	-1.970	< 0.001
Normal	723(51.90)	445(53.04)	278(50.18)	11.835	0.019
Mild	207(14.86)	131(15.61)	76(13.72)		
Moderate	266(19.10)	166(19.79)	100(18.05)		
Severe	105(7.54)	51(6.08)	54(9.75)		
Extremely severe	92(6.60)	46(5.48)	46(8.30)		
Anxiety symptoms	9.88(7.66)	9.95(7.58)	9.78(7.79)	0.430	0.436
Normal	598(42.93)	349(41.60)	249(44.95)	4.527	0.339
Mild	158(11.34)	106(12.63)	52(9.39)		
Moderate	301(21.61)	186(22.17)	115(20.76)		
Severe	147(10.55)	87(10.37)	60(10.83)		
Extremely severe	189(13.57)	111(13.23)	78(14.08)		
Stress symptoms	12.56(8.61)	12.62(8.52)	12.47(8.75)	0.31	0.188
Normal	871(62.53)	528(62.93)	343(61.91)	1.689	0.793
Mild	196(14.07)	121(14.42)	75(13.54)		
Moderate	205(14.72)	116(13.83)	89(16.06)		
Severe	103(7.39)	62(7.39)	41(7.40)		
Extremely severe	18(1.29)	12(1.43)	6(1.08)		
Significant others social support	18.31(5.86)	18.12(5.80)	18.60(5.95)	-1.490	0.516
Family social support	17.39(5.72)	17.15(5.52)	17.76(6.01)	-1.960	0.009
Friends social support	19.99(5.12)	19.72(5.19)	20.4(4.99)	-2.410	0.158
School bullying	11.1(3.96)	11.8(4.24)	10.04(3.22)	8.320	< 0.001
Internalized homophobia	32.62(7.87)	35.1(7.66)	28.86(6.59)	15.700	0.003

### Measures

The survey encompassed questions covering demographics information, school bullying, internalized homophobia, social-psychological resources, and mental health. The selected measures are widely recognized in the field.

Demographics: Demographic data collected included age, sex, sexual orientation, race/ethnicity, education, and area of residence.

### LGB victimization

Internalized homophobia: Measured using the Internalized Homophobia Scale [18], an empirically validated 11-item self-administered scale (e.g., “If I were a heterosexual, I would be happier”). Participants rated the frequency of experienceing such thoughts and feelings on a 5-point scale ranging from 1 (strongly disagree) to 5 (strongly agree). The Cronbach’s alpha for this scale in the current study was 0.82.

School bullying: Assessed using the Supporting LGBT lives subscale of the school bullying [19], consists of 8 items (e.g., “Isolate you because you are or think you are a member of LGBT”). Items were scored on a scale from 1 (no) to 5 (very frequent), with higher scores indicating more frequent experiences of school bullying. The Cronbach’s alpha for this scale in the current study was 0.86.

### Social–psychological resources

Social support: Assessed using the Multidimensional Scale of Perceived Social Support (MSPSS) [20], which comprises 12-item assessing subjective social support from family, friends, and significant others (e.g., “My friends really try to help me”). Responses were rated on a 7-point scale from 1 (completely disagree) to 7 (completely agree). In the current study, the Cronbach’s alpha for family, friends, and significant others were 0.87, 0.92, 0.91, respectively.

### Mental health problems

Depression, Anxiety, and Stress: Measured using the Depression Anxiety Stress Scales (DASS-21) (simplified Chinese version) [21], a 21-item self-report measure of depression, anxiety, and stress (e.g., “I felt my life had no meaning whatsoever.”). Each subscale consists of 7 items, with responses rated on a 4-point scale (ranging from 0 to 3 points, 0 = “Did not apply to me at all”, 1 = “Applied to me to some degree, or some of the time”, 2 = “Applied to me to a considerable degree or a good part of the time”, and 3 = “Applied to me very much or most of the time”). Severity ratings for each subscale were defined as follows: Depressive Symptoms (normal 0–4, mild 5–6, moderate 7–10, severe 11–13, extremely severe 14 +), Anxiety Symptoms (normal 0–3, mild 4–5, moderate 6–7, severe 8–9, extremely severe 10 +), and General Stress (normal 0–7, mild 8–9, moderate 10–12, severe 13–16, extremely severe 17 +). Higher scores indicated higher levels of anxiety, depression, or stress. The scale has good face validity and test–retest reliability [22, 23]. The Cronbach’s alphas for depressive, anxiety, and stress subscales in this study were 0.87, 0.82, and 0.84, respectively.

### Analytic plan

Descriptive statistics were employed to characterize the demographics and affective symptoms (depression, anxiety, and stress) for the entire sample and by gender. Chi-square tests were used to compare male and female individuals regarding demographics and affective symptoms. Independent-sample t-tests were conducted to explore gender-based differences in minority stress variables (internalized homophobia, school bullying, and social support).

Hierarchical multiple regression analyses were performed to investigate the effect of minority stress on affective symptoms, controlling for other variables affecting affective symptoms. These included tobacco use, suicide attempts, self-rated esteem, self-rated health, and demographic factors. Prior to running multiple regressions, the assumptions required for regression analysis were assessed and confirmed to be met without necessitating data adjustments.

Structural equation model (SEM) was employed to examine whether perceived social support moderate the mediated relationships between school bullying with affective symptoms via internalized homophobia. SEM enables the simultaneous testing of relationships between all variables and underlying constructs. This approach offers several advantages, including the identification of direct and indirect effects, and the corresponding standard errors and obtain indices of overall model fit. Full-information maximum-likelihood estimation analyses ware used to retain as much data as possible [24].

There are various difficulties in using latent variables in a structural equation analysis of interactions. To find the impacts of interactions, fixed factor coefficients and error variances must first be subjected to nonlinear constraints. Even though every variable that makes up the interaction term has a normal distribution, it can still be challenging to claim that the indicators of the interaction term do as well. In an effort to overcome these limitations, Ping's two-step method (1996), which is less dependent on the use of nonlinear constraints, was used in this study to confirm the impact of interactions [25].

The data in this study were at the ordinal level; therefore, the SEM assumption of multivariate normality was not possible. Additionally, the Mardia’s coefficients for multivariate kurtosis in each model was > 3, indicating significant multivariate non-normality in the data. As a result, the Bollen–Stine bootstrap *P* procedure was used to adjust model fit and parameter estimates to accommodate the lack of multivariate normality [26, 27].

**Fig. 1:**
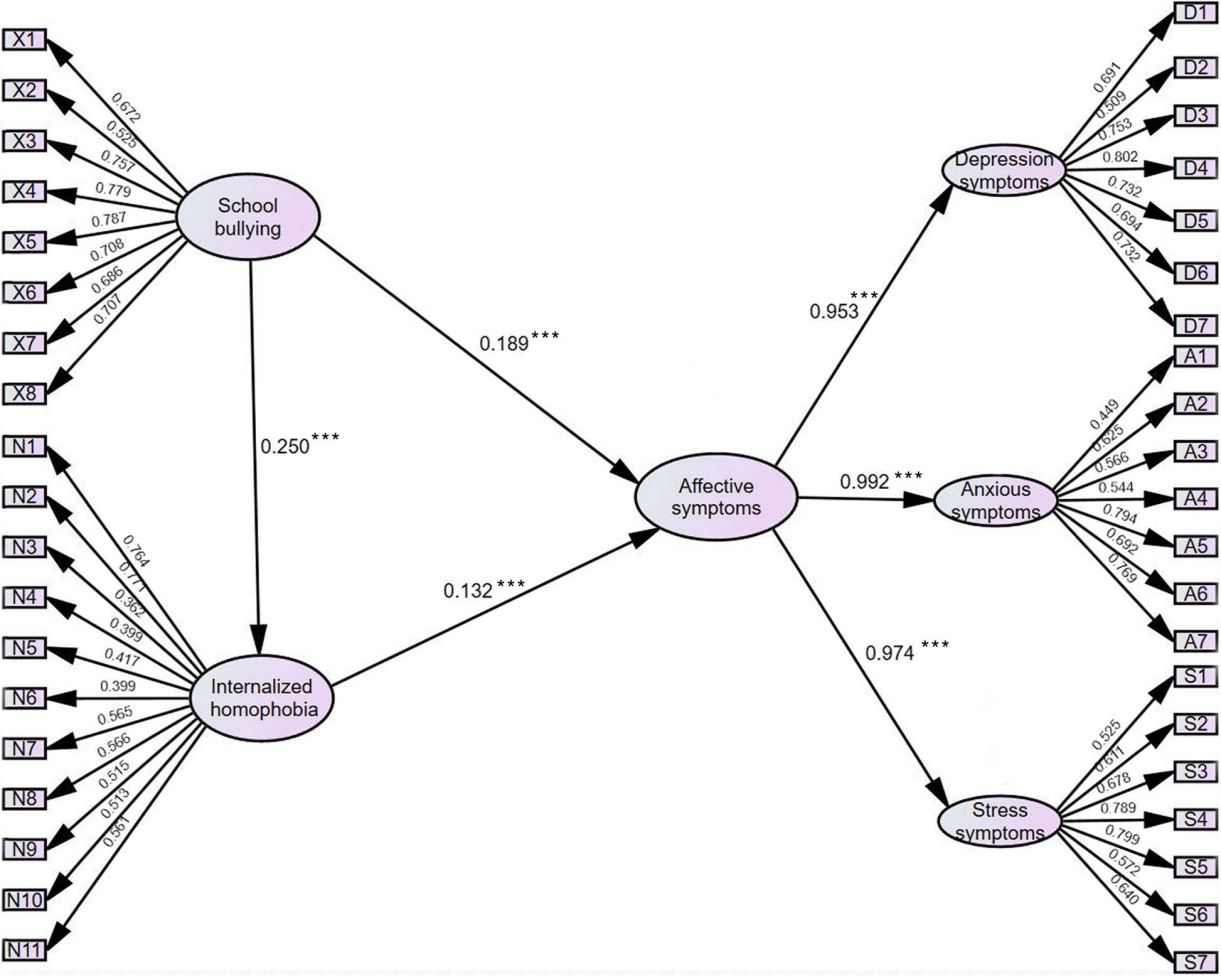
Path model and standardized path coefficients for prediction of health outcomes *P < 0.05. ** *P* < 0.01. *** *P* < 0.001

Model fit to the sample data was evaluated through a two-step procedure [28]. First, a measurement model was tested with all relevant paths left free to vary. Then, the hypothesized structural path model was examined, wherein all hypothesized paths shown in Fig. 1 were estimated freely. Modification indices were inspected for significant areas of model misfit, and the model was adjusted accordingly and rerun. Model fit was assessed using goodness of fit index (GFI), comparative fit index (CFI), Tucker–Lewis Index (TLI), and root-mean-square error of approximation (RMSEA). Indicators of acceptable model fit are considered to be a GFI, CFI and TLI > 0.90, RMSEA < 0.06 [29, 30]. The analyses were conducted using SPSS (version 22) and Amos (Version 26). A *P*-value of less than 0.05 was considered statistically significant.

### Patient and public involvement

The design and conduct of this study were informed through discussion with the LGBT members. Five LGBT members were part of the study steering group and advised on a lay summary of our study findings.

## Results

### Preliminary analyses

Significant differences were observed between male and female individuals in various aspects, including sexual orientation (χ^2^ = 132.545, *P* < 0.001), urban or rural residence (χ^2^ = 58.187, *P* < 0.001), education (χ^2^ = 16.654, *P* = 0.002), major (χ^2^ = 55.646, *P* < 0.001), parents' marital status, mother's and father's education level, monthly expenses, tobacco use, alcohol use, and suicide attempts (*P* < 0.025, see Table 1). Among females, a significantly higher proportion identified as bisexual, resided in urban areas, be freshmen, major in humanities and social sciences, have parents with unstable marital status, and had higher educated mothers and fathers compared to their male counterparts. Additionally, a higher proportion of females reported monthly expenses exceeding RMB 2000, as well as higher rates of tobacco and alcohol use, and no suicide attempts. Independent samples t-tests indicated that females had significantly lower levels of school bullying (t = 8.320, *P* < 0.001) and internalized homophobia (t = 15.700, *P* = 0.003), as well as higher levels of family support (t = -1.960, *P* = 0.009). Table 2 displays the intercorrelations among minority stress and mental health variables.

**Table 2:** Descriptive statistics and intercorrelations of the variables

	1	2	3	4	5	6	7	8
1. Depression symptoms	—							
2. Anxiety symptoms	0.945^***^	—						
3. Stress symptoms	0.930^***^	0.965^***^	—					
4. Significant others social support	-0.370^***^	-0.269^***^	-0.294^***^	—				
5. Family social support	-0.421^***^	-0.313^***^	-0.327^***^	0.554^***^	—			
6. Friends social support	-0.365^***^	-0.312^***^	-0.280^***^	0.862^***^	0.596^***^	—		
7. School bullying	0.207^***^	0.249^***^	0.188^***^	-0.091^**^	-0.109^***^	-0.159^***^	—	
8. Internalized homophobia	0.156^***^	0.167^***^	0.203^***^	-0.124^***^	-0.046	-0.143^***^	0.250^***^	—
Range	0–40	0–38	0–40	4–28	4–28	4–28	8–26	13–57
Mean	10.58	9.88	12.56	18.31	17.39	19.99	11.10	32.62
SD	8.90	7.66	8.61	5.86	5.72	5.12	3.96	7.87
α	0.87	0.82	0.84	0.91	0.87	0.92	0.86	0.86

^*^p < 0.05; ^**^p < 0.01; ^***^p < *0.001*

### Gender disparities in affective symptoms

Around half of the LGB students (48.1%) met the criteria for depression symptoms, with 14.9% classified as mild, 19.1% as moderate, 7.5% as severe, and 6.6% as extremely severe. For anxiety symptoms, the prevalence was 57.1%, with 11.3% mild, 21.6% moderate, 10.6% severe, and 13.6% extremely severe. Regarding stress symptoms, 37.5% met the criteria, with 14.1% mild, 14.7% moderate, 7.4% severe, and 1.3% extremely severe.

Table 1 demonstrates that females were more likely to meet the criteria for depression symptoms than male, as indicated by both in χ^2^-tests (χ^2^ = 11.835, *P* = 0.019) and t-tests (t = -1.970, *P* < 0.001). However, no statistically significant differences were observed between genders in terms of anxiety and stress symptoms.

### Associations of sexual minority stress and social support with mental health

Table 3 presents the results of regression models examining the associations between social support, sexual minority stress, and mental health. After adjusting for demographics and health-related behaviors, social support from significant others was negatively related to depressive symptoms (β = -0.106, *P* = 0.003) and stress symptoms (β = -0.095, *P* = 0.014). However, it was not significantly associated with anxiety symptoms (β = 0.023, *P* = 0.537). Social support from family was negatively related to depressive symptoms (β = -0.182, *P* < 0.001), anxiety symptoms (β = -0.104, *P* < 0.001), and stress symptoms (β = -0.157, *P* < 0.001). Social support from friends was negatively related to anxiety symptoms (β = -0.131, *P* = 0.001), but not significantly associated with depressive symptoms (β = -0.053, *P* = 0.157) or stress symptoms (β = 0.008, *P* = 0.840). School bullying was associated with higher levels of depressive symptoms (β = 0.103, *P* < 0.001), anxiety symptoms (β = 0.123, *P* < 0.001), and stress symptoms (β = 0.104, *P* < 0.001). Internalized homophobia was associated with higher levels of depressive symptoms (β = 0.098, *P* < 0.001), anxiety symptoms (β = 0.113, *P* < 0.001), and stress symptoms (β = 0.139, *P* < 0.001).

**Table 3:** Effect of demographics, health-related behavior, and minority Stress on depressive symptoms, anxiety symptoms, and stress symptoms

	Depressive symptoms		Anxiety symptoms		Stress symptoms	
	B(SE)	β	B(SE)	β	B(SE)	β
Block 1: Demographics						
Age(years, ref: ≤ 20)	-1.411(0.595)	-0.079^*^	-1.365(0.534)	-0.088^*^	-1.355(0.612)	-0.078^*^
Gender(ref: male)	1.712(0.473)	0.094^***^	0.32(0.424)	0.02	0.435(0.487)	0.025
Sexual orientation(ref: Gay/lesbian)	0.697(0.448)	0.036	1.095(0.401)	0.066^**^	0.895(0.461)	0.048
Ethnicity (ref: Han)	-1.067(0.759)	-0.031	-0.836(0.68)	-0.028	-1.197(0.78)	-0.036
Urban or rural areas(ref: Urban)	0.592(0.489)	0.032	1.012(0.438)	0.064^*^	-0.009(0.503)	-0.001
Education(ref: Master and above)						
Freshmen	0.082(0.92)	0.004	1.299(0.824)	0.074	0.416(0.946)	0.021
Sophomore	-0.507(0.89)	-0.024	0.583(0.798)	0.032	-0.111(0.916)	-0.006
Junior	0.374(0.77)	0.018	1.138(0.69)	0.064	0.799(0.792)	0.04
Senior	0.394(0.807)	0.016	0.847(0.723)	0.041	0.192(0.83)	0.008
Major(ref: Medical)						
Humanities and Social sciences	0.291(0.652)	0.013	0.686(0.585)	0.04	-0.226(0.671)	-0.017
Science, engineering and agriculture	0.887(0.64)	0.048	0.774(0.573)	0.048	-0.067(0.658)	-0.008
Way of living(ref: Living alone outside school)						
School dormitory	-2.91(1.258)	-0.109^*^	-2.741(1.128)	-0.119^*^	-2.481(1.294)	-0.097
Share with others outside school	-3.734(1.362)	-0.128^**^	-3.362(1.221)	-0.133^**^	-3.318(1.402)	-0.118^*^
Parents' marital status(ref: Divorced or bereavement)						
Stability	-0.12(0.573)	-0.005	-0.992(0.513)	-0.05	-1.059(0.589)	-0.048
Instability	-0.104(1.024)	-0.003	-1.129(0.918)	-0.032	-1.037(1.054)	-0.026
Mother's education level(ref: Middle school and below)	0.222(0.504)	0.012	0.109(0.451)	0.007	0.656(0.518)	0.038
Father's education level(ref: Middle school and below)	0.91(0.506)	0.050	0.765(0.454)	0.049	0.329(0.521)	0.019
Monthly expense (ref: 3001 ~)						
≤ 1000	0.011(1.045)	0.000	-0.843(0.936)	-0.032	-0.659(1.075)	-0.022
1001 ~	-0.328(0.825)	-0.018	-0.784(0.74)	-0.049	-0.529(0.849)	-0.029
2001 ~	-0.345(0.887)	-0.015	-0.259(0.795)	-0.013	0.09(0.912)	0.004
Block 2: Health-related behavior						
Tobacco use(ref: yes)	-1.542(0.751)	-0.046^*^	-1.876(0.673)	-0.066^**^	-1.589(0.773)	-0.049^*^
Alcohol use(ref: yes)	0.234(0.402)	0.013	0.22(0.36)	0.014	0.618(0.413)	0.035
Suicide attempts(ref: yes)	-2.646(0.873)	-0.069^**^	-2.096(0.782)	-0.064^**^	-1.365(0.898)	-0.037
Self-rated esteem(ref: Disagree)						
Agree	-6.351(1.715)	-0.224^***^	-3.445(1.537)	-0.141^*^	-2.912(1.764)	-0.106
Neutral	-4.354(1.797)	-0.145^*^	-2.449(1.61)	-0.095	-2.091(1.848)	-0.072
Self-rated health(ref: Unhealthy)						
Healthy	-10.409(1.239)	-0.500^***^	-10.569(1.11)	-0.590^***^	-10.054(1.274)	-0.499^***^
Fair	-3.836(1.266)	-0.177^**^	-5.041(1.134)	-0.270^***^	-3.871(1.302)	-0.184^**^
Block 3: Sexual minority Stress						
Social support						
Significant others	-0.161(0.055)	-0.106^**^	0.03(0.049)	0.023	-0.139(0.056)	-0.095^*^
Family	-0.283(0.043)	-0.182^***^	-0.139(0.039)	-0.104^***^	-0.236(0.045)	-0.157^***^
Friends	-0.093(0.066)	-0.053	-0.196(0.059)	-0.131^**^	0.014(0.067)	0.008
School bullying	0.232(0.053)	0.103^***^	0.238(0.047)	0.123^***^	0.227(0.054)	0.104^***^
Internalized Homophobia	0.111(0.028)	0.098^***^	0.11(0.025)	0.113^***^	0.152(0.028)	0.139^***^

*p < 0.05, ** *p* < 0.01, *** *p* < 0.001

### Confirmatory factor analysis

Latent variables were constructed for social support, encompassing subjective social support from family, friends, and significant others, and for mental health, comprising depression, anxiety, and stress. The latent factors were allowed to freely correlate in the measurement model. The model demonstrated an acceptable fit: Bollen-Stine χ^2^_(1262)_ = 1523.781,* P* < 0.05, GFI = 0.963, CFI = 0.0993, TLI = 0.993, RMSEA = 0.012. Factor loadings for the indicators of each latent variable exceeded 0.627 and were all statistically significant (all *Ps* < 0.001). Moreover, the correlations among social support, school bullying, internalized homophobia, and mental health within the measurement model were statistically significant (all *Ps* < 0.001).

### A mediation model of internalized homophobia between school bullying and mental health

SEM also demonstrated an acceptable model fit: Bollen-Stine χ^2^_(1068)_ = 899.05, *P* < 0.05, GFI = 0.967, CFI = 0.994, TLI = 0.993, RMSEA = 0.013. The final model revealed that school bullying was positively associated with internalized homophobia. Additionally, both school bullying and internalized homophobia were directly associated with mental health problems (Fig. 1).

Using bias-corrected bootstrapping with 5000 samples for tests of direct and indirect effects, school bullying and internalized homophobia were significantly directly (β = 0.225, *P* < 0.001) and indirectly associated with mental health (β = 0.039, *P* < 0.001), suggesting partial mediation.

To assess the stability of the structural equation model, a multi-group structural equation model was employed to analyze whether the model differed among various groups, such as gender, sexual orientation, age, urban or rural residence, and tobacco use. The results indicated that the model exhibited a certain degree of stability among different groups (results not presented).

### Social support will moderate the strength of the mediated relationships between school bullying with affective symptoms via internalized homophobia

To examine whether social support moderated the strength of the mediated relationships between school bullying and affective symptoms via internalized homophobia, an interaction term was created using Ping’s (1996) two-step approach. A moderated mediation model was constructed, and the model also demonstrated an acceptable fit: Bollen-Stine χ^2^(1068) = 736.943, *P* < 0.05, GFI = 0.968, CFI = 0.993, TLI = 0.992, RMSEA = 0.014.

**Fig. 2:**
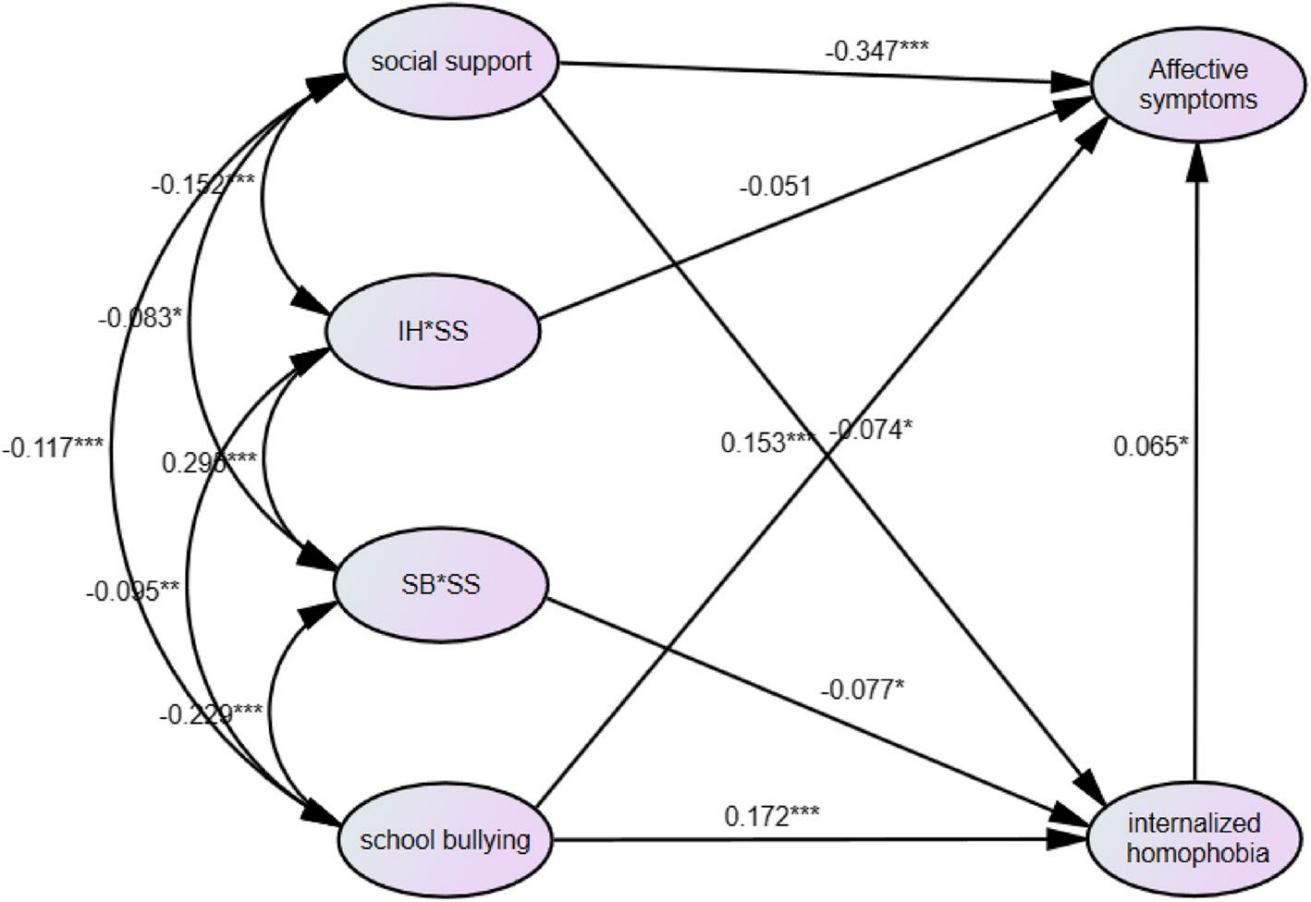
Mediated moderation path results for affective symptoms *P < 0.05. ** *P* < 0.01. *** *P* < 0.001

As shown in Fig. 2, only the path where the interaction term moves toward internalized homophobia was found significant (β = -0.077, *P* = 0.040). The path from school bullying toward internalized homophobia was also significant (β = 0.172, *P* < 0.001). Regarding the path from the mediating variables toward affective symptoms—a dependent variable—the path from internalized homophobia toward affective symptoms was significant (β = 0.065, *P* = 0.023). Moreover, the direct path taken by school bullying leading to affective symptoms was significant (β = 0.153, *P* < 0.001). This implies that even after the main effect is controlled in the full measurement model that includes the interaction effect, internalized homophobia has a mediation effect between school bullying and affective symptoms. Table 4 provides the significance of the path coefficients of each variable.

**Table 4:** Moderated mediation paths' coefficients

Paths	Standardized	Unstandardized			*P*
	Estimate	Estimate	S.E	C.R	
School bullying → Internalized homophobia	0.172	0.648	0.124	5.227	< 0.001
SBSS → Internalized homophobia	-0.077	-0.074	0.036	-2.052	0.040
Social support → Internalized homophobia	-0.074	-0.018	0.007	-2.424	0.015
Social support → Affective symptoms	-0.347	-0.039	0.003	-11.404	< 0.001
Internalized homophobia → Affective symptoms	0.065	0.030	0.013	2.281	0.023
School bullying → Affective symptoms	0.153	0.266	0.052	5.164	< 0.001
IHSS → Affective symptoms	-0.051	-0.004	0.002	-1.775	0.076

*SBSS* school bullying x social support, *IHSS* internalized homophobia x social support

Table 5 illustrated the conditional effects at incremental levels of social support, ranging from low levels to high levels of social support. Notably, simple slope tests indicated that the indirect effect of school bullying on affective symptoms through internalized homophobia was significant when social support was at low (β = 0.049, 95% CI 0.012–0.103) and moderate (β = 0.019, 95% CI 0.003–0.042) levels.

**Table 5:** Conditional indirect effect of school bullying on affective symptoms at special level of social support

Moderator Variable	Level	Estimate	Bias-Corrected 95%CI			Percentile 95%CI		
			Lower	Upper	*P*	Lower	Upper	*P*
Social Support	high level(-1SD)	0.003	-0.006	0.030	0.322	-0.010	0.021	0.620
	Average level	0.019	0.003	0.042	0.017	0.002	0.040	0.023
	int_low level(1SD)	0.049	0.012	0.103	0.008	0.010	0.100	0.013

## Discussion

LGB individuals face substantial health disparities [31], a reality also mirrored among LGB students [32, 33]. In our study, a diverse group of Chinese LGB undergraduates exhibited notably high levels of depression (48.1%), anxiety (57.1%), and stress (37.5%). However, it's essential to recognize that LGB students’ mental health may be influenced by their country of residence. Different nations have distinct culture orientations along the collectivistic-individualistic spectrum and diverse approaches to LGBTQ rights [34]. In China, psychological well-being is closely tied to the satisfaction of needs within the collective [1]. Consequently, the activation of social resources, like social support, can have a profound impact on mental health. While extensive national studies have documented adverse health outcomes among LGB individuals, there has been limited research among Chinese undergraduates. Our study is, to our knowledge, the first to explore minority stressors and mental health (specifically, depression, anxiety, and stress symptoms) in a large, national sample of Chinese LGB undergraduates. The research not only delves into the unique roles of school bullying and internalized homophobia but also incorporated a moderator, social support, into the stress-health model. It’s worth noting that previous discussions of minority stress have primarily focused, both theoretically and empirically, on its influence on mental health [31].

Our findings underscore the detrimental effects of school bullying and internalized homophobia on the lives of sexual and gender minority students, particularly in the context of psychological well-being [7, 32]. Those who experience rejection from friends or unfair treatment from family members, or even strangers, are at increased risk of mental health problems such as depression, anxiety, and stress [10]. These, in turn, can lead to reduced life satisfaction [35]. Importantly, social support from family and significant others was associated with lower levels of depression and stress symptoms [36]. Furthermore, social support from family and friends was linked to lower levels of anxiety symptoms. This is especially significant because LGB individuals face a heightened risk of affective symptoms due to discrimination, health issues, or the loss of a family member or close friend, all of which can generate stress for a student [37]. College students are in a transitional phase, balancing academic and professional pursuits, which is also a high-risk period for the development of mental health issues [13, 38]. This is the study to address the intersection of these identities and the resulting additive effects of unique forms of discrimination on mental health of LGB undergraduates. While this model cannot pinpoint the exact causes of mental health problems, it highlights that bullying and internalized homophobia are predictors of subsequent mental health disorders [7, 39] and are distressing in their own right. School bullying can leave victims feeling isolated and helpless, while internalized homophobia can lead to deep self-denial. Meyer [5] believed that internal sexual minority stress has the greatest and more direct impact on the mental health of sexual minorities. However, this study found that school bullying has a more substantial impact on mental health than internalized homophobia. LGB-based school bullying may have statistically overshadowed internalized homophobia with a larger standardized regression weight because LGB students can experience LGB-based school bullying from their own LGB students, in addition to discrimination based on sexual or gender identity they may experience from individuals inside and outside the LGB community [32]. Consequently, LGB students may feel compelled to conceal their sexual orientation in an effort to fit in, a strategy that, although protective in some instances, carries cognitive and behavioral burdens that result in serious psychological consequences, including depression and anxiety [1, 40].

Experiencing LGB-based school bullying has an additive effect on the social support experienced by undergraduates, which, in turn, impacts the mental health and quality of life of LGB students, and may even be associated with physical health problems in adulthood [15, 41]. Due to the sensitivity of sexual minorities, LGB college students may feel isolated or excluded when they experience sexual minority stress like school bullying and internalized homophobia. This can lead to perceive the availability of social support as lower than it actually is and result in an inaccurate subjective assessment of their level of social support from family, friends, and significant others [42]. This perception can significantly affect their mental health and perceived social support. Social support, both theoretically and empirically, is widely recognized as an essential factor in the well-being of LGB individuals [4, 43], helping them find or maintain meaning in life, especially in the face of oppression.

Consistent with the Psychological Mediation Framework [31], our findings provide supportive evidence for the indirect effect of distal minority stress experiences (school bullying) on affective symptoms (depression, anxiety, and stress symptoms) via internalized homophobia. This finding aligns with a study conducted among young gay men [44]. Moreover, social support was found to moderate the path between school bullying and internalized homophobia. This is to say, social support reduced the effect of school bullying on sexually internalized homophobia. In other words, individuals with both higher school bullying and lower social support were more likely to internalized homophobia [45]. This is significant because internalized homophobia, as a proximal stressor, occurs when individuals have negative feelings and homophobia attitudes towards themselves and others who are part of the sexual minority [5]. In Chinese culture, which is socially conservative and less tolerant of homosexuality [46], many people, including those who routinely provide support to friends, family members, and other in their daily lives, may hold prejudice views toward homosexuality. Perceived social support, seem as “emotional support”, is associated with successful coping in the face of stressors [47]. High social support may facilitate effective coping, such as dealing with school bullying, to maintain low levels of internalized homophobia. Interestingly, our findings did not reveal a moderating effect of social support in the relationship between internalized homophobia and affective symptoms [46, 48]. This could be due to the fact that the majority of respondents were enrolled in school and received “virtually” social support from their family members through long-distance contacts, which may have diminished the perceived strength of social support.

Additionally, research suggests that support from other LGB individuals may have a more substantial impact on mental health than support from heterosexual individuals [49]. As our findings indicate, interventions that encourage LGB individuals to actively utilize social resources may be especially beneficial.

### Implications

The impact of LGB-based school bullying on internalized homophobia in LGB students, and thereby mental health, moderated by social support, is a potentially important area for intervention based on the findings. Schools must pay close attention to the minority stress experienced by LGB students and incorporate resource mobilization into interventions aimed at preventing and addressing mental health issues. Indeed, results indicate the relevance of psychosocial interventions that address minority stress and the environment, such as LGB-affirmative cognitive behavioral therapy. This therapy offers students the opportunities to learn coping strategies related to the stress of sexual minority status and has been shown to significantly reduces depressive and anxiety symptoms [50]. Additionally, cognitive-behavioral therapy like “Effective Skills to Empower Effective Men” have been successful in improves the mental and sexual health of Chinese young men who have sex with men (YMSM) [51]. Meanwhile, clinicians or school psychologists may choose to focus on helping LGB students reduce their negative self-perceptions and attitudes (i.e., internalized homophobia) and reevaluate their coping mechanisms for school bullying. Given the connection between minority stress, resources, and mental health, treatments that take the social environment into account may provide valuable insight for LGB students.

Furthermore, our study shows that social support plays a decreasingly moderating role in the indirect association between school bullying and internalized homophobia. Future psychological intervention should not only focus on the sexuality minority but also on colleges, family members, and friends. Colleges should promote anti-stigma, anti-discrimination, and anti-bullying messages more vigorously, offer multi-gender education, enhance social support for LGB students, assist them in coming to terms with their sexual orientation, reduce the likelihood of internalizing distal stress, and support their psychological well-being. Parents and friends should remain vigilant regarding college students' lifestyle choices and mental health. When students experience negative emotions, timely psychological counseling should be offered to address affective disorde. Implementing multifaceted interventions like these could ultimately help mitigate the mental health problems faced by students, including depression, anxiety, and stress symptoms.

#### Limitations

While this study possesses several strengths, including a substantial sample size and the use of nationwide data from 142 colleges and universities in 31 provinces in China, it also has some notable limitations: 1. Self-Reported Data: The data used in this study relies on self-reported responses, which may be susceptible to participant misunderstanding or biased response. Participants might underreport or overreport certain experiences or emotions. 2. Internet-Based Survey: The survey was conducted online, which has both advantages and disadvantages. While it can enhance accessibility for hard-to-reach populations, such as those who conceal their sexuality, it's challenging to determine how many people viewed the recruitment materials or if there were systematic differences between participants and non-participants. 3. Sample Representativeness: It's important to acknowledge that the sample may not be fully representative of the entire population of LGB college students, primarily because many LGB individuals keep their sexual orientation secret. Thus, the exact number of LGB students in the population remains unknown. 4. Cross-Sectional Design: This study utilized cross-sectional data, which cannot establish causal relationships between minority stress, social support, and mental health. It provides valuable insights into associations but cannot determine the direction of causality.

## Conclusions

In conclusion, this study highlights that LGB college students in China, especially those assigned female at birth, are more likely to experience depression, anxiety, and stress symptoms. We found that the link between school bullying and affective symptoms is partially mediated by internalized homophobia. Moreover, our results demonstrate that low social support exacerbates the impact of school bullying on internalized homophobia, subsequently increasing the risk of affective symptoms among LGB college students.. Effective strategies to improve the mental health status of LGB college students should involve collaborative efforts from society, educational institutions, and families.

### Supplementary Information

**Additional file 1: Appendix Table A1. **Measurement Model.

## Data Availability

The datasets generated or analysed during the current study are not publicly available to maintain the privacy of the individuals’ identities. The dataset supporting the conclusions is available upon request to the corresponding author.

## Abbreviations

LGB: Lesbian, Gay, and Bisexual
DASS-21: The Depression Anxiety Stress Scales
SEM: Structural equation modelling
