# Supplementary material for: Minority stress, social support and mental health among lesbian, gay, and bisexual college students in China: a moderated mediation analysis
Source: BMC Psychiatry. 2023 Oct 13;23:746. doi: 10.1186/s12888-023-05202-z (PMC10576333; doi:10.1186/s12888-023-05202-z)
Supplement: Supplementary file 1 — Additional file 1: Appendix Table A1. Measurement Model. [file 12888_2023_5202_MOESM1_ESM.docx]

| Appendix Table A1 Measurement Model | | | | | |  |  |  |  |
| --- | --- | --- | --- | --- | --- | --- | --- | --- | --- |
| Latent variable | Indicator | Standardized | Unstandardized | | | *P* | SMC | AVE | CR |
|  |  | *B* | *B* | *S.E.* | *Z* |  |  |  |  |
| Social support | Significant others | 0.899 | 1.710 | 0.103 | 16.552 | <0.001 | 0.808 | 0.705 | 0.874 |
| Social support | Friend | 0.956 | 1.833 | 0.107 | 17.054 | <0.001 | 0.915 |  |  |
| Social support | Family | 0.627 | 1.000 |  |  |  | 0.393 |  |  |
| Affective symptoms | Depression symptoms | 0.959 | 1.000 |  |  |  | 0.919 | 0.948 | 0.982 |
| Affective symptoms | Anxiety symptoms | 0.989 | 0.681 | 0.044 | 15.508 | <0.001 | 0.978 |  |  |
| Affective symptoms | Stress symptoms | 0.972 | 0.740 | 0.041 | 18.049 | <0.001 | 0.944 |  |  |
| School bullying | Item 8 | 0.708 | 1.000 |  |  |  | 0.501 | 0.500 | 0.888 |
| School bullying | Item 7 | 0.687 | 0.836 | 0.035 | 23.827 | <0.001 | 0.472 |  |  |
| School bullying | Item 6 | 0.709 | 0.632 | 0.026 | 24.284 | <0.001 | 0.503 |  |  |
| School bullying | Item 5 | 0.789 | 0.871 | 0.032 | 27.065 | <0.001 | 0.622 |  |  |
| School bullying | Item 4 | 0.778 | 1.358 | 0.052 | 25.931 | <0.001 | 0.605 |  |  |
| School bullying | Item 3 | 0.756 | 1.292 | 0.051 | 25.308 | <0.001 | 0.571 |  |  |
| School bullying | Item 2 | 0.524 | 1.242 | 0.069 | 17.968 | <0.001 | 0.274 |  |  |
| School bullying | Item 1 | 0.671 | 1.189 | 0.052 | 22.810 | <0.001 | 0.45 |  |  |
| Internalized homophobia | Item 11 | 0.560 | 1.000 |  |  |  | 0.314 | 0.378 | 0.801 |
| Internalized homophobia | Item 10 | 0.487 | 0.811 | 0.055 | 14.816 | <0.001 | 0.237 |  |  |
| Internalized homophobia | Item 9 | 0.473 | 0.808 | 0.056 | 14.347 | <0.001 | 0.223 |  |  |
| Internalized homophobia | Item 8 | 0.520 | 0.930 | 0.060 | 15.581 | <0.001 | 0.271 |  |  |
| Internalized homophobia | Item 7 | 0.525 | 0.951 | 0.060 | 15.887 | <0.001 | 0.275 |  |  |
| Internalized homophobia | Item 2 | 0.820 | 1.578 | 0.088 | 17.919 | <0.001 | 0.672 |  |  |
| Internalized homophobia | Item 1 | 0.808 | 1.574 | 0.088 | 17.917 | <0.001 | 0.652 |  |  |
| Significant others | Item 1 | 0.791 | 1.000 |  |  |  | 0.625 | 0.709 | 0.907 |
| Significant others | Item 2 | 0.880 | 1.212 | 0.032 | 37.526 | <0.001 | 0.775 |  |  |
| Significant others | Item 5 | 0.853 | 1.212 | 0.034 | 35.606 | <0.001 | 0.728 |  |  |
| Significant others | Item 10 | 0.842 | 1.177 | 0.034 | 34.808 | <0.001 | 0.709 |  |  |
| Friend | Item 6 | 0.882 | 1.000 |  |  |  | 0.778 | 0.741 | 0.920 |
| Friend | Item 7 | 0.887 | 1.059 | 0.022 | 47.822 | <0.001 | 0.786 |  |  |
| Friend | Item 9 | 0.851 | 0.982 | 0.023 | 42.166 | <0.001 | 0.725 |  |  |
| Friend | Item 12 | 0.822 | 0.971 | 0.025 | 39.374 | <0.001 | 0.675 |  |  |
| Family | Item 11 | 0.613 | 1.000 |  |  |  | 0.376 | 0.631 | 0.870 |
| Family | Item 8 | 0.824 | 1.431 | 0.059 | 24.099 | <0.001 | 0.68 |  |  |
| Family | Item 4 | 0.902 | 1.545 | 0.063 | 24.470 | <0.001 | 0.813 |  |  |
| Family | Item 3 | 0.810 | 1.279 | 0.054 | 23.483 | <0.001 | 0.656 |  |  |
| Depression symptoms | Item 3 | 0.693 | 1.000 |  |  |  | 0.48 | 0.500 | 0.873 |
| Depression symptoms | Item 5 | 0.509 | 0.807 | 0.045 | 17.995 | <0.001 | 0.259 |  |  |
| Depression symptoms | Item 10 | 0.752 | 1.324 | 0.051 | 26.057 | <0.001 | 0.565 |  |  |
| Depression symptoms | Item 13 | 0.803 | 1.238 | 0.044 | 27.828 | <0.001 | 0.644 |  |  |
| Depression symptoms | Item 16 | 0.731 | 1.096 | 0.043 | 25.452 | <0.001 | 0.534 |  |  |
| Depression symptoms | Item 17 | 0.693 | 0.993 | 0.041 | 24.001 | <0.001 | 0.481 |  |  |
| Depression symptoms | Item 21 | 0.731 | 1.164 | 0.046 | 25.209 | <0.001 | 0.534 |  |  |
| Anxiety symptoms | Item 2 | 0.447 | 1.000 |  |  |  | 0.2 | 0.415 | 0.828 |
| Anxiety symptoms | Item 4 | 0.626 | 1.151 | 0.075 | 15.258 | <0.001 | 0.392 |  |  |
| Anxiety symptoms | Item 7 | 0.564 | 1.092 | 0.075 | 14.521 | <0.001 | 0.319 |  |  |
| Anxiety symptoms | Item 9 | 0.541 | 1.474 | 0.104 | 14.154 | <0.001 | 0.293 |  |  |
| Anxiety symptoms | Item 15 | 0.795 | 1.684 | 0.101 | 16.640 | <0.001 | 0.633 |  |  |
| Anxiety symptoms | Item 19 | 0.692 | 1.487 | 0.094 | 15.854 | <0.001 | 0.479 |  |  |
| Anxiety symptoms | Item 20 | 0.769 | 1.748 | 0.106 | 16.468 | <0.001 | 0.592 |  |  |
| Stress symptoms | Item 1 | 0.525 | 1.000 |  |  |  | 0.276 | 0.444 | 0.845 |
| Stress symptoms | Item 6 | 0.610 | 1.252 | 0.073 | 17.184 | <0.001 | 0.372 |  |  |
| Stress symptoms | Item 8 | 0.677 | 1.548 | 0.084 | 18.335 | <0.001 | 0.458 |  |  |
| Stress symptoms | Item 11 | 0.790 | 1.719 | 0.087 | 19.813 | <0.001 | 0.625 |  |  |
| Stress symptoms | Item 12 | 0.801 | 1.795 | 0.089 | 20.086 | <0.001 | 0.641 |  |  |
| Stress symptoms | Item 14 | 0.571 | 1.263 | 0.077 | 16.467 | <0.001 | 0.326 |  |  |
| Stress symptoms | Item 18 | 0.639 | 1.363 | 0.077 | 17.674 | <0.001 | 0.408 |  |  |
